# Clinical Comparison of Distal Pancreatectomy with or without Splenectomy: A Meta-Analysis

**DOI:** 10.1371/journal.pone.0091593

**Published:** 2014-03-28

**Authors:** Zhigang He, Daohai Qian, Jie Hua, Jian Gong, Shengping Lin, Zhenshun Song

**Affiliations:** Department of General Surgery, Shanghai Tenth People's Hospital, Tongji University of Medicine, Shanghai, China; National Taiwan University, Taiwan

## Abstract

**Objective:**

A distal pancreatectomy has routinely been used for removing benign/borderline malignant tumors of the body and tail of the pancreas; however, controversy exists whether or not the spleen should be saved. Therefore, we conducted this meta-analysis for comparing the clinical outcomes of patients who underwent distal pancreatectomy with or without splenectomy.

**Methods:**

A literature research from the databases of Medline, Embase, and Cochrane library was performed to evaluate and compare the clinical outcomes between spleen-preserving distal pancreatectomy (SPDP) and distal pancreatectomy with splenectomy (DPS). Pooled odds ratio (OR) and weighted mean difference (WMD) with 95% confidence interval (95% CI) were calculated using fixed-effects or random-effects models.

**Results:**

Eleven non-randomized controlled studies involving 897 patients were selected to satisfy the inclusion criteria; 355 patients underwent SPDP and 542 patients underwent DPS. Compared with DPS, SPDP required a shorter hospital stay (WMD = 1.16, 95% CI = −2.00 to −0.31, P = 0.007), and had a lower incidence of intra-abdominal abscesses (OR = 0.48, 95% CI = 0.27 to 0.83, P = 0.009). In addition, spleen infarctions occurred in SPDP, most of which involved use of the Warshaw method for preserving the spleen. There were no differences between the SPDP and DPS groups with respect to operative time, operative blood loss, requirement for blood transfusion, pancreatic fistulas, thromboses, post-operative bleeding, wound infections and re-operation rates.

**Conclusion:**

SPDP should be performed due to the benefits of the immune system and quick post-operative recovery. It is also essential to preserve the splenic artery and vein. Large randomized controlled trials are further needed to verify the results of this meta-analysis.

## Introduction

A distal pancreatectomy (DP) is the preferred procedure when resecting benign and borderline malignant tumors of the body and tail of the pancreas. The spleen is usually resected by three methods (laparoscopic, open, or laparoscopic conversion techniques) because it is rather close to the tail of the pancreas. How can one think that the spleen is unimportant in our body and does not put our lives at risk? Through the investigation and follow-up of patients who have undergone splenectomies, poor prognoses are associated with overwhelming post-splenectomy infections (OPSIs), hypercoagulability, and hematologic malignancies [1]–[3]. Therefore, more and more surgeons have begun to realize the importance of salvaging the spleen during distal pancreatectomy for non-malignant diseases.

Friedrich Trendelenburg [4] is credited as the first surgeon to resect a solid tumor of the tail of the pancreas in 1882 at the University of Bonn in Germany. Spleen-preserving distal pancreatectomy (SPDP) has been widely performed since first described by Mallet-Guy and Vachon in 1943 [5]. In the SPDP, the splenic artery and vein are spared by separating and ligating the pancreatic tributaries. Warshaw et al [6] introduced the other method of preserving the spleen by saving the short gastric and gastroepiploic vessels. Both techniques are feasible with minimally invasive approaches, which are well-described and safe to perform [6], [7].

Theoretically, the spleen should be preserved [8], simply due to the favorable role of regulating the balance of the hematologic and immune systems; however, many authors suggest that splenic preservation is more time-consuming, which may lead to greater blood loss, and higher incidences of pancreatic fistulas and subphrenic abscesses [9]–[12]. Based on above considerations, we undertook this meta-analysis to compare the clinical outcomes between SPDP and distal pancreatectomy with splenectomy (DPS).

## Methods

### Article Search

The analysis of previous studies was conducted in accordance with the Preferred Reporting Items for Systematic Reviews and Meta-Analyses (PRISMA) guidelines. A comprehensive search was carried out to include all trials that compared the clinical outcomes between SPDP and DPS before September 2013 using the key words (distal pancreatectomy, spleen preservation, spleen-preserving, splenic preservation, and splenectomy) in the Medline, Embase, and Cochrane Library electronic databases. Reference lists of all the retrieved articles were manually searched for additional studies. The search was restricted to articles in English.

### Inclusion Criteria

For inclusion in this meta-analysis, a study had to fulfill the following criteria: 1) compare SPDP and DPS among patients who underwent distal pancreatectomy for benign or borderline malignant diseases; 2) report on at least one of the clinical outcome measures mentioned below and provide the standard deviation of the mean for the continuous outcomes of interest (or provide sufficient data to calculate the standard deviation); 3) clearly report the indications for the SPDP and DPS groups; and 4) in the case that dual (or multiple) studies were reported by the same institution and/or authors, the study of higher quality or the most recent publication was included in the analysis. Abstracts, letters, editorials, expert opinions, reviews without original data, case reports, and studies without control groups were excluded.

### Outcomes of Interest

The interesting clinical outcomes included operative outcomes (operative time, operative blood loss, and number of patients requiring blood transfusion) and post-operative outcomes (pancreatic fistulas, wound infections, intra-abdominal abscesses, post-operative bleeding, thromboses, re-operation, and hospital stay).

### Statistical Analysis

This meta-analysis was performed using Review Manager (RevMan) software (version 5.0.2). We analyzed the dichotomous variables by estimating the odds ratio (OR) with a 95% confidence interval (95% CI), and continuous variables were analyzed using the weighted mean difference (WMD) with a 95% CI. The pooled effect was calculated using fixed or random effects models. Heterogeneity was measured using the Q-test, and heterogeneity was evaluated using I^2^, which can be interpreted as the percentage of the total variation between studies that can be attributable to heterogeneity rather than chance. The scale of I^2^ values ranged between 0% and 100%, with higher values denoting a greater degree of heterogeneity. A p value <0.05 was considered significant. Generation of a funnel plot and the Egger p-value allowed determination of the potential publication bias of the included studies. The Newcastle-Ottawa Scale (NOS) was used to assess the quality of the studies.

## Results

### Included Studies

**Figure 1:**
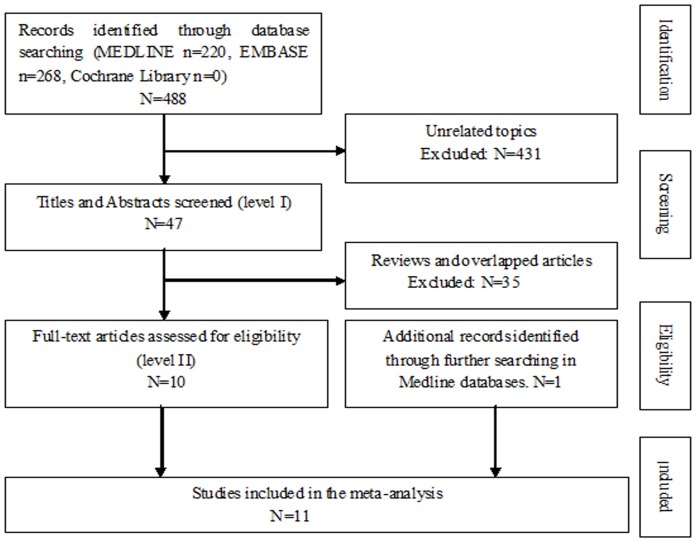
Flow diagram of our method of evidence.

Four hundred eighty-eight articles were found, as follows: Medline, n = 220; Embase, n = 268; and Cochrane Library, n = 0. In addition to using the keywords to find eligible studies, one study was identified by further identification of potentially relevant studies in Medline.11 eligible studies published between 1989 and 2013 were finally identified according to our predefined selection criteria (**Fig 1**) [13]–[25]. The studies included a total of 897 patients, as follows: SPDP group, n = 355 (39.58%); and DPS group, n = 542 (60.42%). Of these patients, 143 (18.67%) had undergone laparoscopic surgery (Lap) and 607 (81.33%) had undergone conventional open surgery (Open), and 2 patients who undergone laparoscopic surgery were converted to Open were still considered Lap. Four studies were conducted in the US, one in Japan, one in Korea, two in France, and three in China. The sample size of each study varied from 21 to 259 patients. The study characteristics and patient demographics are summarized in **Table 1**. In these 11studies, the patients in the two groups were matched according to age, gender, duration of follow-up, histopathologic diagnosis, and the operative methods. In addition, the Newcastle-Ottawa Scale (NOS) was utilized to assess the quality of each study included in our meta-analysis, which is indicated in **Table 2**.

**Table 1:** Baseline characteristics of included trials. (N or n =  number; y =  year; m =  month; F =  Female; M =  male; Lap =  laparoscopic surgery; Open =  open surgery; NR =  not reported; * all the patients in both groups; SPDP =  spleen-preserving distal pancreatectomy; DPS =  distal pancreatectomy with splenectomy).

Study	Number (N)		Gender(F/M)		Age (y)		Follow up (m)		Operations (N)		Warshaw (n/N)
	SPDP	DPS	SPDP	DPS	SPDP	DPS	SPDP	DPS	SPDP	DPS	
Benoist et al. 1998 France	15	25	11/4	8/17	43	51	34	30	Open	Open	1/15
Carrèr et al. 2006 France	38	38	26/12	21/17	47	55	65	65	Open	Open	38/38
Choi et al. 2012 Korea	40	32	26/14	24/8	48.5	50.2	26.3	34.2	Lap	Lap	11/40
Feng et al. 2013 China	78	82	22/56	20/62	64.5	62.2	43.5	42.2	Open	Open	NR
MA et al. 2011 China	13	13	8/5	8/5	*66	*66	NR	NR	Open	Open	0/13
Mekeel et al. 2011 USA	10	24	8/2	15/9	58	58	19	29	Lap	Lap	0/10
Richardson et al. 1989 USA	11	10	NR	NR	43.9	44.5	NR	NR	Open	Open	1/11
Rodríguez et al. 2006 USA	74	185	55/19	131/54	54.5	58	NR	NR	Open	Open	74/74
Shoup et al. 2002 USA	46	79	*82/43	*82/43	*64	*64	21	21	Open	Open	NR
Yamaguchi et al. 2001 Japan	9	38	5/4	21/17	53.9	56.2	NR	NR	Open	Open	NR
Zhao et al. 2012 China	21	16	15/6	11/5	48	48	35	35	Lap	Lap	6/21

**Table 2:** Newcastle-Ottawa Scale (NOS) assessment of the quality of the studies.

Study	Selection (MAX 4)	Comparability (MAX 2)	Exposure (MAX 3)	All (MAX 9)
Benoist et al. 1998 France	3	2	2	7
Carrèr et al. 2006 France	4	2	2	8
Choi et al. 2012 Korea	4	2	2	8
Feng et al. 2013 China	3	2	2	7
MA et al. 2011 China	3	2	1	6
Mekeel et al. 2011 USA	4	2	2	8
Richardson et al. 1989 USA	3	2	1	6
Rodríguez et al. 2006 USA	4	2	1	7
Shoup et al. 2002 USA	4	2	2	8
Yamaguchi et al. 2001 Japan	3	2	1	6
Zhao et al. 2012 China	4	2	2	8

### Operative Outcomes

**Figure 2:**
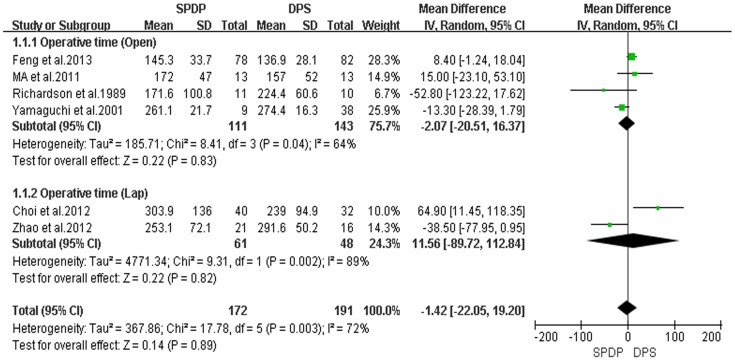
Operative time (min) (IV, Inverse variance, M-H, Mantel-Haenszel, CI, Confidence Interval; SD, Standard deviation).

**Figure 3:**
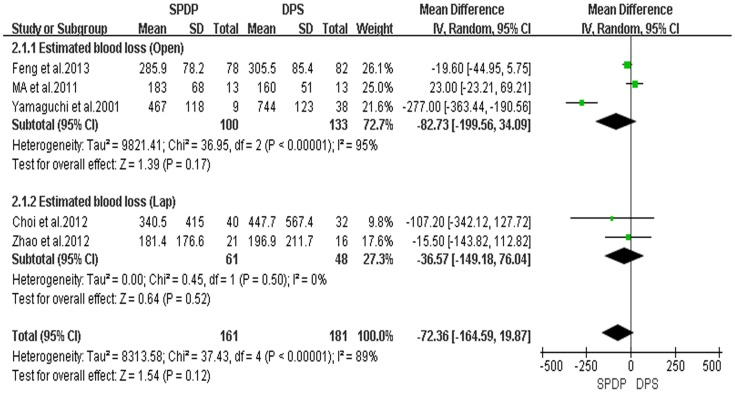
Estimated blood loss (IV, Inverse variance, M-H, Mantel-Haenszel, CI, Confidence Interval; SD, Standard deviation).

**Figure 4:**
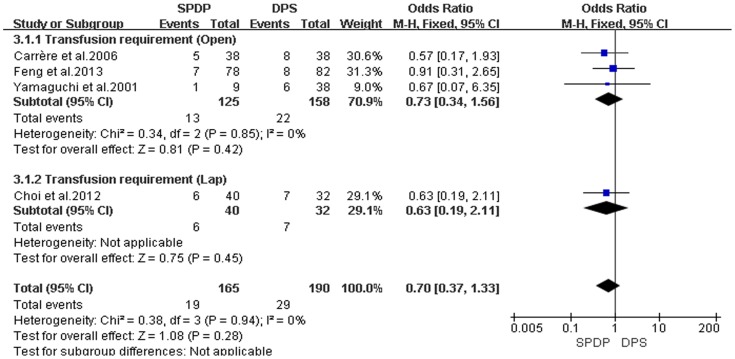
Transfusion requirement (IV, Inverse variance, M-H, Mantel-Haenszel, CI, Confidence Interval; SD, Standard deviation).

In these 11 retrospective controlled studies, 6 reported the operative time; and the result of the meta-analysis showed no difference in the operative time between the SPDP and DPS groups (WMD = −1.42, 95% CI = −22.05 to 19.20, P = 0.89); This finding indicated a significant difference in heterogeneity between studies (I^2^ = 72%, χ^2^ = 17.78, P heterogeneity  = 0.003). Subgroup analysis also revealed no significant difference between SPDP and DPS in the Open and Lap groups (**Fig 2**). Five studies reported the operative blood loss, which did not differ between the two groups when the data were pooled for the SPDP and DPS groups (WMD = −72.36, 95% CI = −164.59 to 19.87, P = 0.12), and subgroup analysis showed the similar statistical outcome, being also associated with a significant difference in heterogeneity between the studies (I^2^ = 89%, χ^2^ = 37.43, P heterogeneity <0.00001; **Fig 3**). Further observation revealed, to some extent, that the heterogeneity affected by the choice of the surgical approach and subgroup analysis also indicated that no statistical difference existed among studies for operative time and operative blood loss in the Lap group. There was no significant difference between the two groups with respect to the number of transfused patients (OR = 0.70, 95% CI = 0.37 to 1.33, P = 0.28); no statistically significant heterogeneity among studies was observed (I^2^ = 0%, χ^2^ = 0.38; P heterogeneity  = 0.94; **Fig 4**).

### Post-operative Outcomes

**Figure 5:**
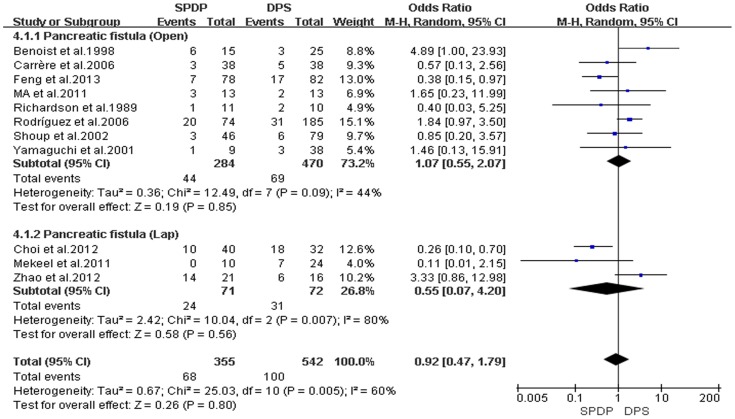
Pancreatic fistula (IV, Inverse variance; M-H, Mantel-Haenszel; CI, Confidence Interval; SD, standard deviation).

**Figure 6:**
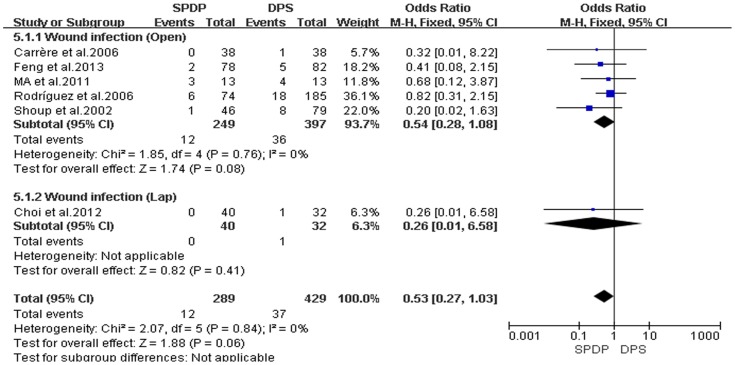
Wound infection (IV, Inverse variance; M-H, Mantel-Haenszel; CI, Confidence Interval; SD, standard deviation).

**Figure 7:**
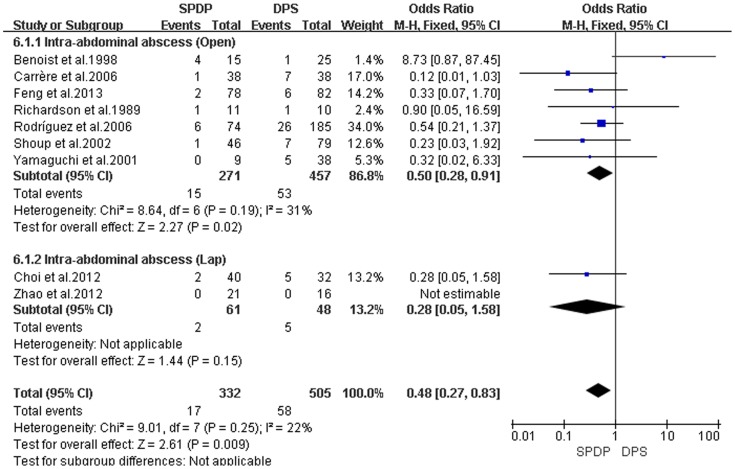
Intra-abdominal abscess (IV, Inverse variance; M-H, Mantel-Haenszel; CI, Confidence Interval; SD, standard deviation).

**Figure 8:**
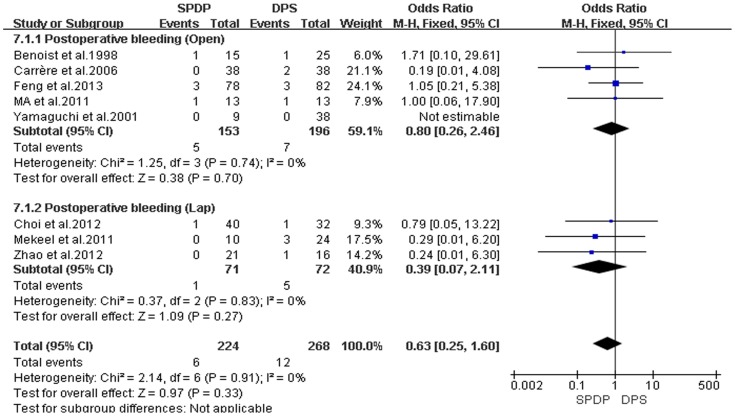
Postoperative bleeding (IV, Inverse variance; M-H, Mantel-Haenszel; CI, Confidence Interval; SD, standard deviation).

**Figure 9:**
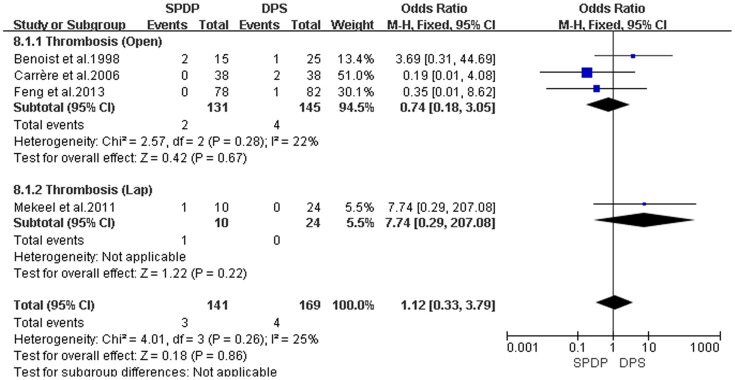
Thrombosis (IV, Inverse variance; M-H, Mantel-Haenszel; CI, Confidence Interval; SD, standard deviation).

**Figure 10:**
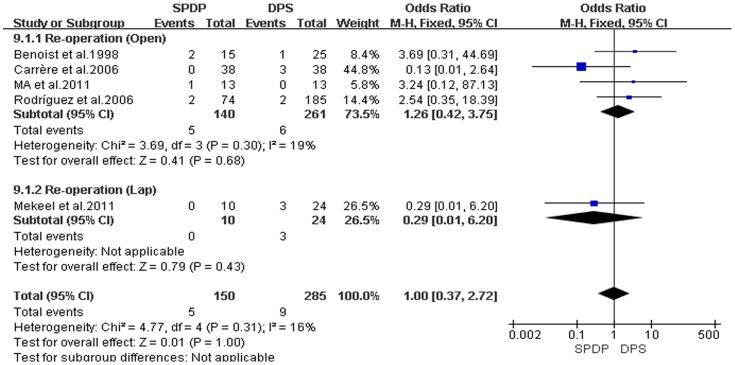
Re-operation (IV, Inverse variance; M-H, Mantel-Haenszel; CI, Confidence Interval; SD, standard deviation).

**Figure 11:**
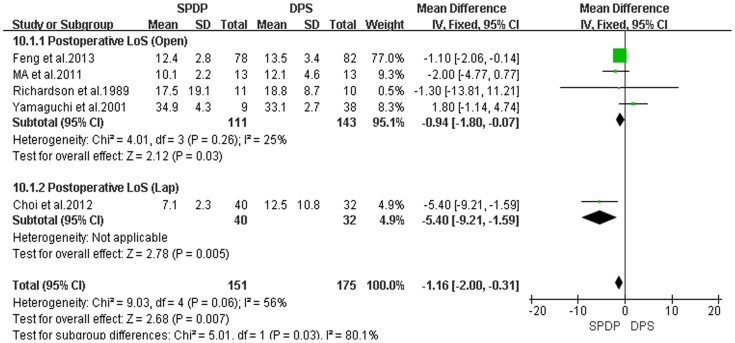
Postoperative LoS (days) (IV, Inverse variance; M-H, Mantel-Haenszel; CI, Confidence Interval; SD, standard deviation).

Pancreatic fistulas, which are a serious complication of pancreatectomies, and all of the included studies compared the pancreatic fistula rate between the SPDP and DPS groups; however, according to our analysis, there was no difference in the incidence of pancreatic fistulas between the two groups(OR = 0.92, 95% CI = 0.47 to 1.79, P = 0.80; **Fig 5**). No significant difference existed regarding the rates of wound infections between the SPDP and DPS groups (OR = 0.53, 95% CI = 0.27 to 1.03, p = 0.06), with no significant heterogeneity (I^2^ = 0%, χ^2^ = 2.07, P heterogeneity  = 0.84; **Fig 6**). Subgroup analysis indicated that the rates of wound infections were comparable between the SPDP and DPS groups whether or not Open or Lap was performed. In contrast, the rates of intra-abdominal abscesses were significantly lower in the SPDP group than the DPS group (OR = 0.48, 95% CI = 0.27 to 0.83, P = 0.009) with a low level of heterogeneity (I^2^ = 22%, χ^2^ = 9.01, P heterogeneity  = 0.25; **Fig 7**). Subgroup analysis also revealed significantly lower rates of intra-abdominal abscesses with SPDP for the Open group. Post-operative bleeding was reported in eight trials. There was no significant difference in the mean post-operative bleeding between groups (OR = 0.63, 95% CI = 0.25 to 1.60, P = 0.33). No evidence of statistically significant heterogeneity was observed (I^2^ = 0%, χ^2^ = 2.14, P heterogeneity  = 0.91; **Fig 8**). Subgroup analysis showed parallel outcomes for the Open and Lap groups. In addition, there was a similar incidence of thrombosis and re-operation (OR = 1.12, 0.33 to 3.79, P = 0.86 and OR = 1.00, 0.37 to 2.72, P = 1.00, respectively; **Fig 9** and **Fig 10**). Low heterogeneity was found among the studies that reported these outcomes as well. Five reports compared the length of hospital stay between the two groups; in 4 studies, the hospital stay was shorter in the SPDP group (WMD = −1.16, 95% CI = −2.00 to −0.31, P = 0.007). Subgroup analysis also showed significantly shorter hospital stays with SPDP in the Open and Lap groups. There was no statistically significant heterogeneity among studies in the Open group (I^2^ = 25%, χ^2^ = 4.01, P heterogeneity  = 0.26; **Fig 11**). The mortality was 0% and 1.09% in the SPDP and DPS groups, respectively (0 vs. 5 deaths), which was reported in 8 trials. Through careful examination of the complete data set, it was not difficult to show that spleen infarction occurred more frequently in the SPDP group using the Warshaw method (two of three cases),Other complications, such as ileuses, post-operative diabetes mellitus, pseudocysts, and cardiac complications, were only reported in a small number studies. Therefore, we did not analyze these data.

### Publication Bias

**Figure 12:**
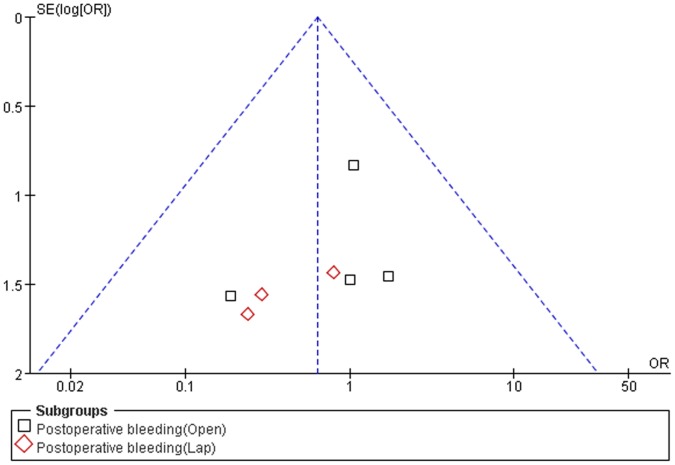
Funnel plots of postoperative bleeding. OR odds ratio; SE (log [OR]): standard error of the natural logarithm of the odds ratio.

Funnel plots were used to evaluate the possibility of publication bias. The shapes of the funnel plots for transfusion requirements, wound infections, intra-abdominal abscesses, post-operative bleeding, thromboses, re-operations, and post-operative LoS did not reveal asymmetry, indicating no evidence of publication bias (**Fig 12**).

## Discussion

The statistical results suggest that spleen-preserving should be done in patients with benign or low-malignant diseases and the spleen artery and vein should be saved. In contrast, spleen salvage should be an alternative method for surgeons when using the Warshaw technique [24].

In this meta-analysis, the data revealed that SPDP can be performed without extending the operative time, increasing operative blood loss and the risk of post-operative complications. Furthermore, the pooled data showed a shorter hospital stay, fewer intra-abdominal abscesses for the SPDP group in comparison with the DPS group. However, spleen infarction occurred more frequently in the SPDP group when using the Warshaw technique. The operative time, blood loss, post-operative bleeding, re-operation, and mortality are key indicators to evaluate whether or not a surgical technique is safe for patients. Indeed, we found that there were no differences in the aforementioned indications between the two groups; Tsiouris and Lee et al. [25], [26] demonstrated that an effort to preserve the adult spleen during distal pancreatectomy is worthwhile, and both Open and Lap methods were used in the SPDP group as well as the DPS group. In addition, the conclusion of the polled comparison studies was consistent with this meta-analysis, with the exception of Benoist et al. [13]. Thus, the spleen should be salvaged if possible when performing a distal pancreatectomy.

There was no significant difference between the two groups in operative outcomes, including operative time and estimated blood loss, and these results are consistent with recent comparative study trials [16, 17, and 21]. Tsiouris et al. [25] reported that there was a tendency towards a longer operative time and increased blood loss in the DPS group; however, our meta-analysis of the pooled data did not confirm the difference.

The post-operative pancreatic fistulas, which are amongst the most serious complications following distal pancreatectomy [27]–[29], were similar in both groups (SPDP: 19.15% versus DPS: 18.45%). The method of pancreatic transaction was similar between the SPDP and DPS groups. In the spleen-preserving laparoscopic distal pancreatectomy (Sp-Lap DP) and laparoscopic distal pancreatosplenectomy (Lap DPS) groups, the pancreas was usually transected using a harmonic scalpel, LiagSure, bipolar cautery, or an Endo GIA stapler [30], whereas in the open spleen-preserving distal pancreatectomy (OSPDP) and open distal pancreatectomy with splenectomy groups, pancreatic transaction was usually performed using an electrocautery blade or Endo GIA stapler. The pancreatic stump was usually oversewn with suture reinforcement using black silk or polypropylene. Overall, whether or not the spleen is preserved, it is vital to deal with the pancreatic stump carefully. Because the pancreas is an endocrine and exocrine organ, and pancreatic trypsin has a strong corrosive effect with the capacity to auto-digest. Therefore, how to avoid pancreatic fistulas is always a challenge for the surgeon. In this meta-analysis we found that all studies had reported the occurrence of pancreatic fistulas. We conclude that the pancreatic fistula is inevitable due to leakage from pinprick sites when stitching the pancreatic stump with suture reinforcement. Small pancreatic fistulas seldom lead to patient mortality. The re-operation rate and mortality in each group were extremely low, as demonstrated in the current study, which is a reflection of the progress in pancreatic surgery.

Salvaging the spleen should favor the immune system and reduce the risk of infections [31]. In this meta-analysis the rates of wound infections were similar between the DPS and SPDP groups, but the SPDP group had a significantly lower rate of intra-abdominal abscesses, which were closely related to the recovery and cost to the patients. The basis for these results is less damage to the patients and the balance of immune system in the SPDP group.

Post-operative bleeding, thrombosis, and re-operation were not significantly different between the two groups; however, the hospital stays were significantly shorter in the SPDP group than the DPS group [12], which benefitted patient recovery and reduced the costs. Moreover, the immediate post-operative course was as important as their lives following the operative procedure, would require surgeons to attach importance to both of them. Considering the current results of splenectomy-related potential complications, this issue should not be overlooked in clinical practice.

The pooled data showed significant difference between the two groups with respect to spleen infarction (SPDP: 2.5% versus DPS: 0%) [31]. Warshaw et al. [6] reported that there is a low incidence of spleen infarction in distal pancreatectomies by saving the short gastric and gastroepiploic vessels, which was subsequently referred to as the Warshaw technique; their follow-up data showed no statistical difference compared to the group in which the spleen artery and vein was saved. Through a detailed examination of all studies in our meta-analysis, we found that 4 studies had referred to spleen infarction with 3 cases, in which 2 cases occurred when using the Warshaw method, whereas one case occurred when the splenic artery and vein was preserved. Although there was no significant difference between the two methods, there was a similar benefit in preserving the splenic artery and vein in our meta-analysis. Large randomized controlled trials are required to compare the incidence of spleen fraction between the two methods of spleen preservation (Warshaw method and preservation of the spleen artery and vein).

Significant heterogeneity was observed with respect to operative time and intra-operative blood loss. The reason for the observed heterogeneity in operative time, intra-operative blood loss, and the length of hospital stay may be variations in the skill of the surgeon and the histopathologic diagnostic differences at different institutions.

However, we also acknowledge certain inherent limitations in the studies included in our meta-analysis that cannot be ignored when interpreting our data. On one hand, all data were non-randomized controlled trials, which lead to less powerful results than trials based purely on randomized patients. However, it is very difficult to conduct a prospective randomized controlled study due to fatal complications and poor compliance. On the other hand, it was not possible that the patient characteristics were completely matched across all of these studies and it was inevitable to cause the heterogeneity between the groups. We applied a random or fixed effect model to consider the variation. If the heterogeneity (I^2^) was >50%, we adopted a random effect model.

In conclusion, distal pancreatectomy with preservation of the spleen is generally feasible, particularly for benign lesions, and is desirable for long-term benefits. Asplenic patients face many problems, including increased risks in post-operative infectious complications, even overwhelming post-splenectomy infections, hypercoagulability, and malignancy [32]. Therefore, splenic salvage should be performed whenever to avert spleen infarction, which is a serious consequence that is associated with mortality. Although there was no difference between the Warshaw and traditional methods, meticulous preservation of the splenic vein and artery is of great concern. Examination of the spleen after completion of a distal pancreatectomy is essential [33]. The clinical evidence which was generated from retrospective non-randomized controlled trials is less persuasive, thus large random controlled trials are needed.

## Supporting Information

Checklist S1 PRISMA checklist.
(DOC)
