# Correction: Clinical Comparison of Distal Pancreatectomy with or without Splenectomy: A Meta-Analysis

**DOI:** 10.1371/journal.pone.0103464

**Published:** 2014-07-17

**Authors:** The PLOS ONE Staff

Figure 1 is incorrect. The authors have provided a corrected version here.

**Figure 1:**
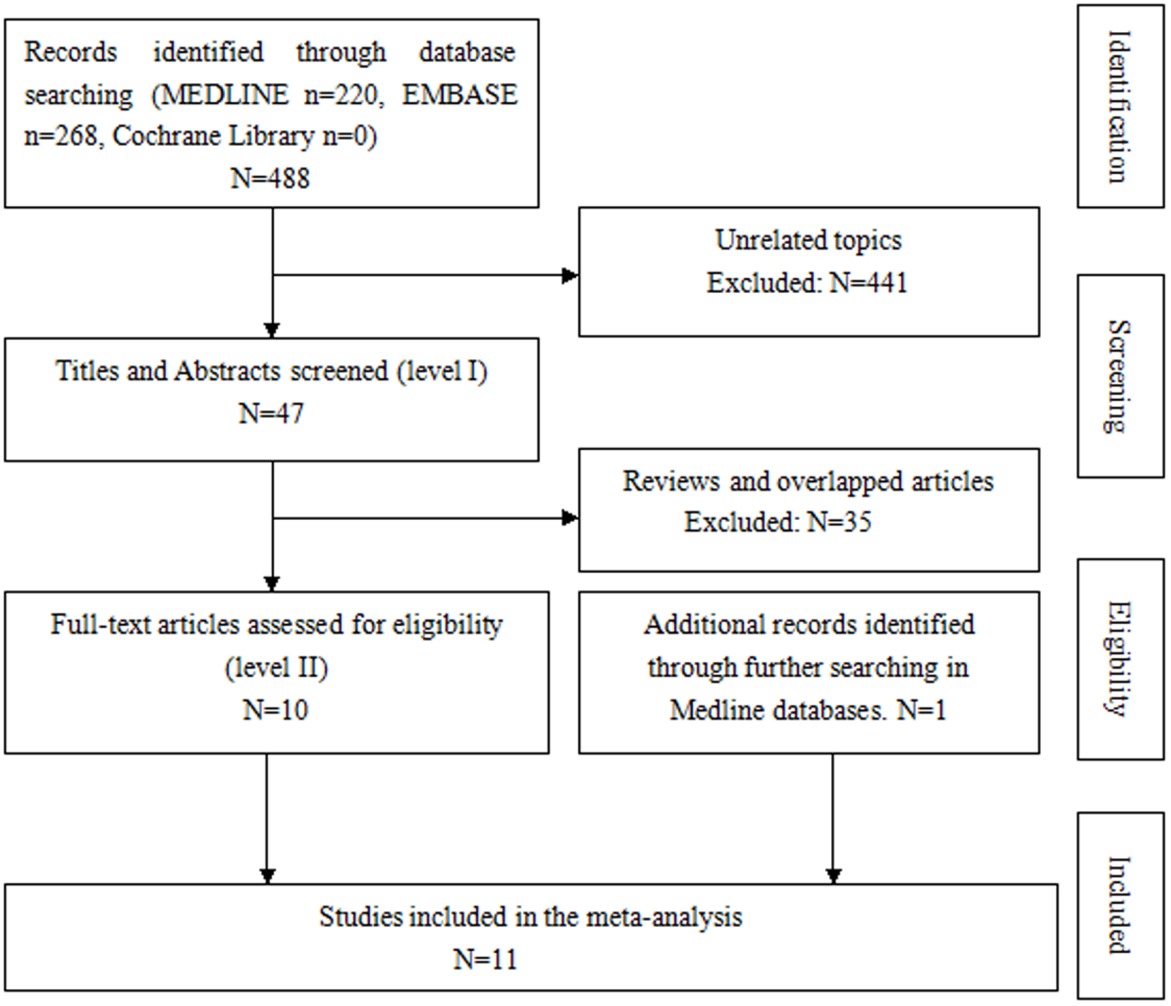
Flow diagram of our method of evidence.
